# Motivation to Decrease Discharge Cost Results in Improper Discharge of Regulated Medical Wastes from Small Clinics: Inspectional and Statistical Evidence in the Tokyo Metropolitan Area

**DOI:** 10.31662/jmaj.2022-0174

**Published:** 2023-04-10

**Authors:** Daisuke Sugimoto, Fumitake Takahashi

**Affiliations:** 1 Tokyo Institute of Technology, Yokohama, Japan; 2 Japan Silver Incorporated, Tokyo, Japan

**Keywords:** medical waste, discharge container, improper disposal, inspection

## Abstract

**Introduction:**

Regulated medical waste (RMW) management, particularly, improper discharges of RMW from small-scale medical institutions (less than 20 sickbeds), has drawn attentions. This study investigated improper discharges of RMW containers from small clinics to analyze improper discharge mechanisms.

**Methods:**

Inspectional survey categorized improper discharges into improper sealing, container deformation, overweight, container contamination, container damaging, etc. The inspection surveys were performed from April 2018 to March 2019. In total, 2364 containers were inspected, which was equivalent to 64317 Litters in container volume and around 13.19 Mg in weight.

**Results:**

About 38% of RMW containers were categorized to improper discharges. They consist mainly of improper sealing (67.0%), container deformation (24.6%), and overweight (6.31%). It was hypothesized that frequent RMW discharges allow short interval of container discharge, which prevents clinic staff from human errors due to forgetting and might reduce improper discharges. However, the inspection results rejected this hypothesis. The survey proposes that improper discharges were likely not sporadic events, which possibly occurred in any clinics, but repeated events in certain clinics. It was also hypothesized that saving discharge cost likely induced overpacking of RMW to containers, particularly larger volume containers, and caused improper sealing, container deformation, and eventually overweight. The inspection results and statistical analyses validated this hypothesis. This study also validated one more hypothesis that large compressive force required for complete sealing might cause improper sealing. The measurement results rejected it. However, they also suggest that gender and age of clinic staff might be partially associated with improper sealing.

**Conclusions:**

Improper discharges of RMW containers seem to be non-random events. Specific clinics likely repeat improper discharges using larger volume containers. It is proposed that decreasing discharge cost induces overpacking of RMW to containers, and causes subsequent problems like container deformation.

## Introduction

**Figure 1.**
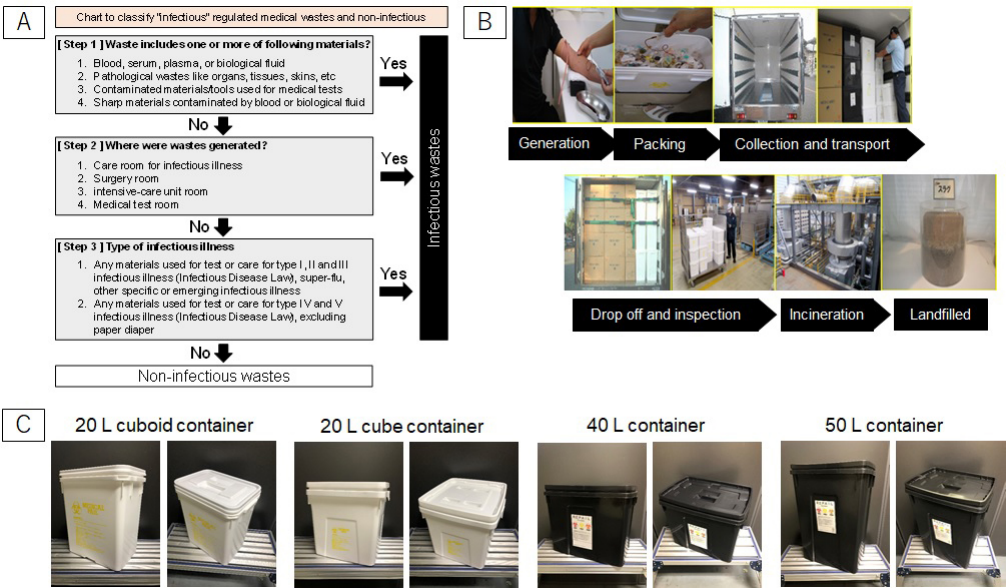
(a) Chart to classify infectious medical waste and non-infectious waste in Japan, (b) RMW disposal flow, (c) Photos of RMW containers (20 L cuboid, 20 L cubic, 40 L, and 50 L volume capacities).

Infectious wastes are defined as ‘*wastes generated from medical institutions, which contain pathogens that are infectious or potentially infectious to humans and wastes wherein pathogens attach or may be attached* ’^(1), (2)^. In Japan, medical wastes generated from medical institutions are classified as industrial waste, specifically into “Specially controlled industrial wastes.” The infectivity of medical wastes is determined according to contamination by pathogens, locations where wastes are generated, and contamination possibility of specific infective disease designated by Infectious Disease Law ^(1)^. Figure 1a shows the determination chart of infectious wastes. Determination charts are often ignored or misunderstood by medical professionals ^(3)^. Due to different strategies or criteria for defining infectious wastes ^(4), (5)^, the lack of standardization makes waste sorting more difficult for healthcare workers, and eventually results in unnecessary infectious waste generation ^(6)^. In fact, high disposal cost of medical wastes is induced by improper segregation of infectious wastes ^(7)^. Careful exclusion of non-infectious wastes is effective to reduce medical waste disposal cost ^(8)^. However, in small-scale clinics, sorting of non-infectious waste is more tedious due to limited labor resources. Furthermore, minimizing regulated medical waste (RMW) discharge costs to make infectious wastes smaller in volume might induce improper sorting.

RMW must be segregated, handled, and discharged properly by medical institutions; it needs to be transported and disposed safely by waste disposal workers to inactivate pathogens and other harmful substances ^(1), (9)^. There are many researches on RMW management ^(10)^, focusing on discharge volume ^(8), (11)^, disposal cost ^(5), (8)^, pollutant emissions from RMW treatment ^(12), (13), (14)^, and others. Owing to strong public concerns on improper treatment and illegal dumping of RMW by transporters and/or disposers, researchers also have investigated those incidents ^(15)^. Meanwhile, improper discharge of RMW by medical institutions, particularly small-scale medical institutions such as clinics, veterinarians, nursing homes, and dental clinics is still uncertain. As described above, it is expected that minimizing disposal costs of RMW might induce improper discharge like overpacking into specific plastic containers. Proper waste discharge is difficult for small-scale medical institutions due to limited labor resources. In this socio-medical context, the authors investigated the appropriateness of RMW discharge from small-scale medical institutions and quantitatively categorized improper disposals. In this article, the authors would propose three hypotheses of improper discharge mechanisms. They would be validated and discussed based on the inspection results.

## Materials and Methods

### RMW disposal flow in Japan

Figure 1b shows the disposal flow of RMW from generation to final landfill in Japan. RMW are put into specific plastic containers, as shown in Figure 1c, sealed completely, and then delivered to treatment facilities (incineration plants). When the containers are sealed once, re-opening the containers before incineration treatment is prohibited. Therefore, there is no way to inspect RMW inside containers after the containers are sealed. It might cause careless or unintended contamination of other regulated materials like mercury used in old clinical thermometers, which should be segregated for necessary treatment, into the containers ^(16)^.

### Inspection survey of RMW container conditions

**Figure 2.**
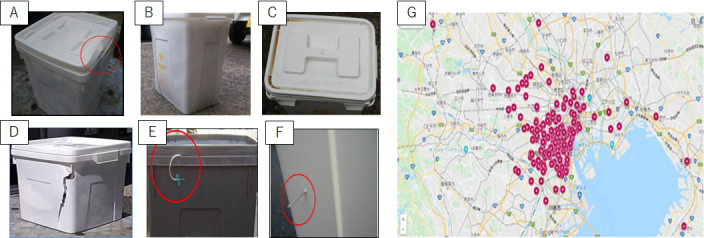
(a-f) Improper discharges of RMW containers [(a) Improper sealing, (b) Container deformation, (c) Container contamination, (d) Container damaging, (e) Overflow, (f) Needle penetration], and (g) Locations of surveyed medical institutions.

The appropriateness of RMW discharge was measured by manual inspection of RMW containers just before the delivery to incineration plants. The inspection was performed by the first author (Daisuke Sugimoto) who managed a company of RMW container collection and transport service (Japan Silver Inc.). Improper discharges were categorized to improper sealing, container deformation, overweight, container contamination, container damaging, overflow, needle penetration, and prohibited content (see Figure 2a, 2b, 2c, 2d, 2e and 2f). The definitions of and the criteria to count these categories are summarized in Table 1. The inspection survey was not informed in advance to the medical institutions in order to avoid any potential biases. The inspection data were collected for one year from April 2018 to March 2019. Figure 2g shows the locations of surveyed small-scale medical institutions, including 131 clinics (less than 20 sickbeds), 12 veterinarians, 6 nursing homes, and 3 dental clinics in Tokyo metropolitan area (152 institutions in total). In total, 2364 containers were inspected, which consists of 2089 containers from clinics, 244 containers from veterinarians, 24 containers from nursing homes, and 7 containers from dental clinics. They were equivalent to 64317 L in container volume (53457 L for clinics, 9700 L for veterinarians, 1020 L for nursing homes, and 140 L for dental clinics) and around 13.19 Mg in weight. Although bulk volumes of RMW were smaller than container volumes, there is no way to measure the bulk volume directly as described above. Therefore, this study assumed that bulk volumes of RMW were equal to container volumes. It should be noted that discussions based on the inspection data includes this limitation. To analyze an impact of waste volume on improper discharge, two-sided Welch’s *t*-test and one-way ANOVA were used to check the significant differences of discharged waste volumes for each category. This study also recorded the number of discharged containers. Therefore, the average numbers of “proper discharge” and “improper discharge” containers were also measured when each small-scale medical institution discharged RMW containers.

**Table 1.** Definition and Counting Criteria of Improper Discharge Categories.

Category	Description	Counting criteria
Improper sealing	Lid of the container is not sealed completely.	When the lid is not sealed at one or more corners, it was counted as improper sealing.
Container deformation	Container or lid is physically deformed, partially bent, or expanded. It shows one or more curved aspects.	When container side surface or lid surface showed curved plane, it was counted as container deformation.
Overweight	The weight of a container exceeds 3 kg for 1 L container, 5 kg for 10 L container, 10 kg for 20 L container, and 15 kg for 40 L and 50 L containers.	When the weight of a container exceeded the weight criteria, it was counted as overweight.
	Note: When RMW containers are incinerated, they are conveyed by belt conveyer (and an elevator) to the incinerator. According to technical specification of manufacturers, the weight load for conveying (and lifting) is recommended 15 kg/container or less.	
Container contamination	The outer surface of a container is contaminated obviously by body fluids like blood, chemicals or medical solutions.	When the surface of a container or a lid shows a spot of obvious color change by contamination, it was counted as container contamination.
Container damaging	Lid or a container has a crack on the surface.	When the surface of a container or a lid shows an obvious crack, it was counted as container damaging.
Overflow	Waste protrudes in-between a lid and a container.	When a part of waste protruded in-between a lid and a container was observed, it was counted as overflow.
Needle penetration	A needle or other sharps is penetrating the surface of a container or a lid.	When a part of sharps including needles penetrating the surface of a lid and a container was observed, it was counted as needle penetration.

### Three hypotheses of improper discharge of RMW containers

The authors propose three hypotheses to explain what causes improper discharge of RMW containers. The first hypothesis is that less actual practices of RMW container discharge induces more improper discharges. Many literatures report the importance of periodic training and/or practices for healthcare workers in terms of RMW management ^(17), (18), (19)^. When small-scale medical institutions discharge RMW containers less frequently, it gives longer interval of container discharge for clinic staff and might induce human errors in disposal steps due to forgetting, incaution, or unawareness. According to this hypothesis, there should be a moderate or strong negative correlation between improper discharge rate and container discharge frequency.

The second hypothesis is that decreasing discharge cost of RMW causes improper discharges as a consequence. Discharge costs are usually determined based on RMW weight for large-scale medical institutions. It should be noted that the costs are often determined based on the number of discharged RMW containers for small-scale medical institutions, not weight. Small-scale medical institutions usually use RMW containers with single volume capacity, not multiple capacities, because of limited storage space for empty containers before use. Additionally, discharge cost is higher for larger capacity containers. When small-scale medical institutions use large capacity containers, they have stronger motivation to discharge RMW using less containers. Therefore, decreasing discharge cost might induce overpacking of RMW. In addition, large capacity containers might also induce overpacking more than small capacity containers. Consequently, overpacking might cause improper sealing and container deformation. According to the second hypothesis, RMW containers discharged improperly should have larger volume capacity than that of properly discharged containers.

The third hypothesis is that necessary compressive force for perfect sealing of container lid is too large. It gives non-negligible difficulty to clinic staff and requires intensive attentions toward proper sealing. If less attention due to busy works and/or limited instruction to proper discharge, it might cause improper sealing. According to this hypothesis, improper sealing would be a random event that is triggered by less attention; particularly, improper sealing would possibly occur in any clinics.

### Compressive force measurement of container lid sealing

In this study, compressive force to seal the lid was measured using a weight balance. To properly and completely seal the lid, a person pushed the lid by hand at each top corner of a container until it sounded as clicked. The maximum weight was recorded when the lid was pushed at each corner. For one container, four maximum weight data were recorded. However, weight data at the last corner were excluded before statistical analysis. At the last corner, non-negligible time gaps between click sound and maximum weight were monitored. Therefore, maximum weight data at the last corner was affected by over-pushing. Maximum weight data were transformed to maximum compressive force (Unit: N) by multiplying gravity acceleration constant (g = 9.807 m/s^2^). In this experiment, four different containers with volume capacity of 20 L, 40 L, and 50 L were tested as shown in Figure 1c. 20 L containers are equilaterally cubic (cube) and vertically long (cuboid). Sample size was 20 persons, who were recruited from students and staffs of Tokyo Institute of Technology, Japan. Their gender and age were summarized in Table 2. For each person, compressive force was measured in triplicate for each container. The average data of each person and each volume container was used for statistical analysis. The impact of gender and age on compressive force to seal the lid was tested by multi-ways ANOVA and Tukey-Kramer multiple comparison test.

**Table 2.** Measurement Results of Compressive Force to Completely Seal a RMW Container.

Gender/Age/Volume	Sample size*	Compressive force for sealing (Unit: newton (N))		
		Mean	Standard deviation	Standard error
Female	36	184.4	79.48	13.25
Male	44	252.4	70.65	10.65
20s	32	229.9	78.55	13.89
40s	28	217.8	97.49	18.42
60s	20	214.5	63.09	14.11
20 L (Cuboid)	20	268.4	85.82	19.19
20 L (Cube)	20	182.6	59.10	13.22
40 L	20	228.8	87.40	19.54
50 L	20	207.5	71.24	15.93

*Total sample size is 80 average data (=20 persons × four different containers)

## Results

### Discharge volume distribution

**Figure 3.**
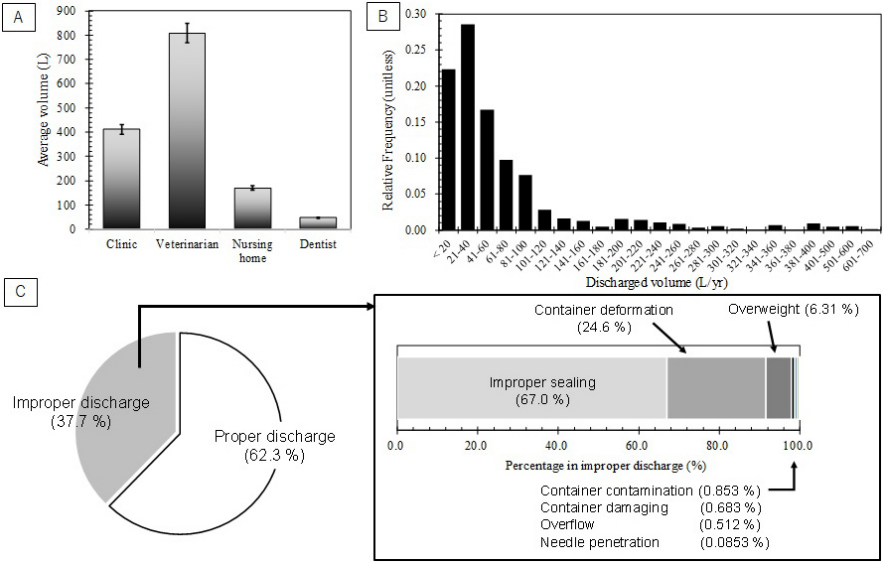
(a) Average discharged container volumes from small-scale medical institutions (clinic, veterinarian, nursing home, and dentist), (b) Relative frequency distribution of average waste volumes, and (c) Inspection results of RMW containers (Proper discharge, improper sealing, container deformation, overweight, container contamination, container damaging, overflow, and needle penetration).

The average volumes of RMW discharged from clinics, veterinarians, nursing homes, and dental clinics are shown in Figure 3a. Veterinarians discharged larger volume of RMW than the others (808 L/year). Dental clinics discharged the smallest volume of RMW (47 L/year). The average discharge volume (411 L/year) of small-scale medical institutions measured in this study is comparable with an estimate (450 L/year) by Takeda and Ozaki ^(20)^. Averagely, small-scale medical institutions discharged 426 L of RMW in a year. The relative frequency distribution of annual discharge volume is shown in Figure 3b. The most frequent discharge volume is 21 to 40 L/year. It suggests that annual frequency of RMW discharge from small-scale clinics is around one to five times. When discharge frequency is lower, interval time to next discharge should be longer. It might weaken instruction effect on proper waste sorting or appropriate container handling for clinic staff ^(21), (22)^.

### RMW discharge appropriateness (container condition inspection)

The results of RMW container inspection are shown in Figure 3c and summarized in Table 3. Based on container number, 62.3% of containers were categorized to proper discharge. The rest 37.7% were improper discharge cases, which consisted of 67.0%, 24.6%, 6.31%, 0.853%, 0.683%, 0.512%, and 0.0853% for improper sealing, container deformation, overweight, container contamination, container damaging, overflow, and needle penetration, respectively (see Figure 3c). Therefore, 96.7% of improper discharge cases were shared by improper sealing, container deformation, and overweight. In this survey, no container that was strongly suspected to include prohibited contents like mercury-containing blood-pressure gauge and gas cylinders. However, even if suspected containers are found, inspection by re-opening suspected containers is prohibited due to legal regulation in Japan.

**Table 3.** Inspection Results of RMW Containers.

	Container number basis		Volume basis (Unit: L)	
	Proper discharge	Improper discharge	Proper discharge	Improper discharge
Container number	1472 (62.3%)	892 (37.7%)	-	-
Container volume	-	-	38607 (60.0%)	25710 (40.0%)
Gross category total*	-	1172 (100%)**	-	37750 (100%)**
Improper sealing	-	785 (67.0%)**	-	22710 (60.2%)**
Container deformation	-	288 (24.6%)**	-	10370 (27.5%)**
Overweight	-	74 (6.32%)**	-	3680 (9.75%)**
Container contamination	-	10 (0.854%)**	-	310 (0.821%)**
Container damaging	-	8 (0.683%)**	-	370 (0.980%)**
Overflow	-	6 (0.512%)**	-	290 (0.768%)**
Needle penetration	-	1 (0.0854%)**	-	20 (0.0530%)**

*Gross category total should be larger than total number/volume because some containers were categorized to two or more types of improper discharge **Percentage within improper cases

### Differences of volume or container number between proper and improper discharges

**Figure 4.**
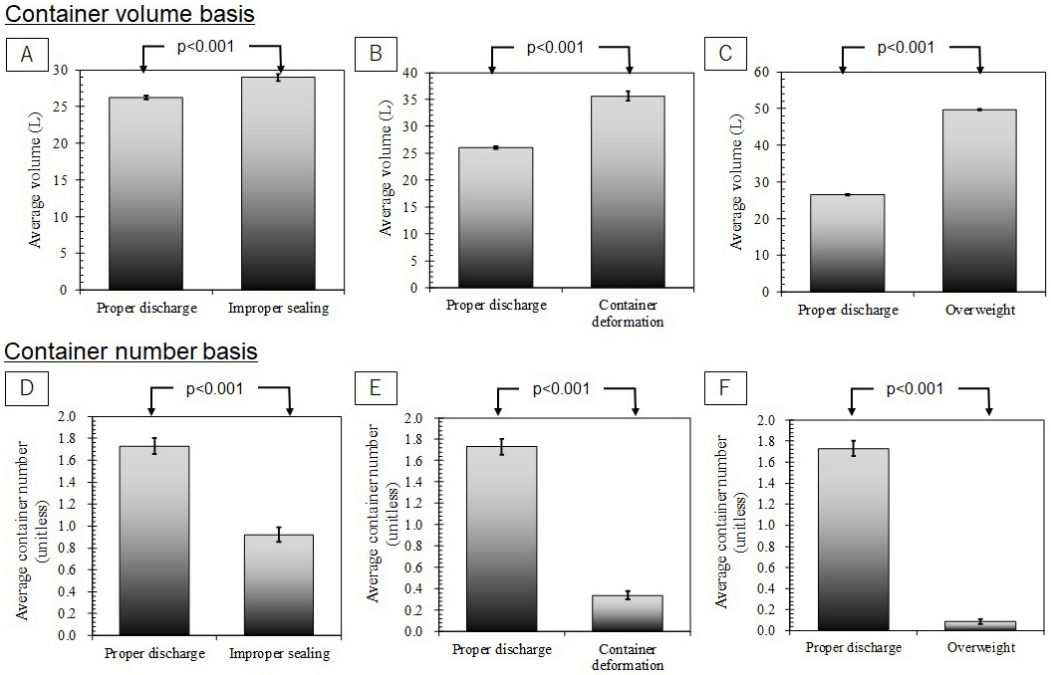
Comparison of “proper discharge” and “improper discharge” containers: (a and d) Improper sealing, (b and e) Container deformation, (c and f) Overweight (Note: [a, b, and c] Container number basis, [d, e, and f] Container number basis, Error bar is standard error, and p value of Welch’s t-test for statistical difference between proper and improper discharges).

As shown in Figure 4a, the average volume capacity of “improper sealing” containers was 11% larger than that of properly discharged containers. This difference was regarded statistically significant (p < 0.001). The average volume of deformed containers was 37% larger than that of proper cases, which is also significant difference (p < 0.001, see Figure 4b). The average volume of “overweight” containers was also significantly 88% larger than that of proper cases (p < 0.001, see Figure 4c). When proper and improper discharge containers are compared based on container numbers, the differences were clearer than those on volume basis. The average numbers of “improper discharge” containers were always significantly smaller than those of proper cases. In the case of improper sealing, it was significantly 50% smaller than that of proper cases (p < 0.001, see Figure 4d). The average numbers were significantly 80% smaller for container deformation (p < 0.001, see Figure 4e) and significantly 95% smaller for overweight (p < 0.001, see Figure 4f), respectively.

### Compressive force required for perfect sealing

**Figure 5.**
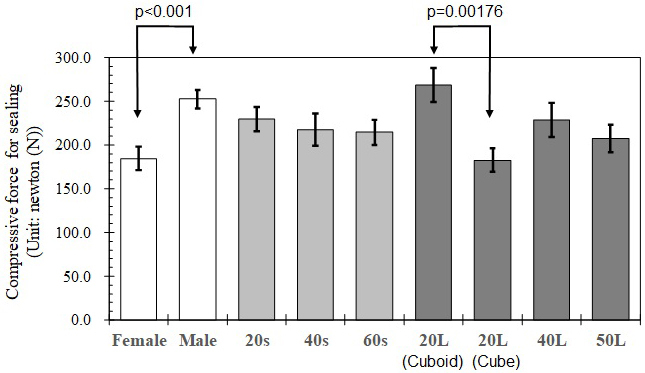
Average compressive force to seal RMW containers in groups of gender, age, and container volume capacity (Note: Error bar is standard error and p value of Welch’s t-test in which significant difference between two categories was identified).

Measurement results of compressive forces to seal the lid are shown in Figure 5 and summarized in Table 2. When the same 20 L volume containers with different geometric configuration (cube; 29 cm × 29 cm × 28 cm, and cuboid; 32 cm × 23 cm × 26 cm) are compared, cuboid 20 L container required significantly larger force for complete sealing than cubic 20 L container (p = 0.00176). Rubber packing ring attached on the lid of cuboid 20 L container is likely conducive to larger compressive force. No packing ring in a lid of cubic 20 L container might result in smaller compressive force. There is no significant difference in compressive forces among different volume containers, except for significant difference between cuboid 20 L container and 50 L container (p = 0.0415). It means that necessary compressive force for complete sealing is almost equal regardless of container volume excluding cuboid 20 L container; whereas, the statistical analysis proposes that the gender and age might induce improper sealing. There is significant difference in measured compressive forces between female and male (p < 0.001). Male gave larger compressive forces than female. In particular, significant differences between genders were found for 20’s (p < 0.001) and 40’s (p = 0.00213). In contrast, no gender difference was found for 60’s. Therefore, if female clinic staff are in charge of RMW container discharges, improper sealing might occur more frequently due to smaller available compressive force. However, further survey is necessary to validate whether smaller compressive force of female is really conducive to improper sealing or not. If so, re-design of container lid for easier sealing might be more effective than instruction or training of clinic staff.

## Discussion

### First hypothesis validation: Does long interval induce improper discharge?

**Figure 6.**
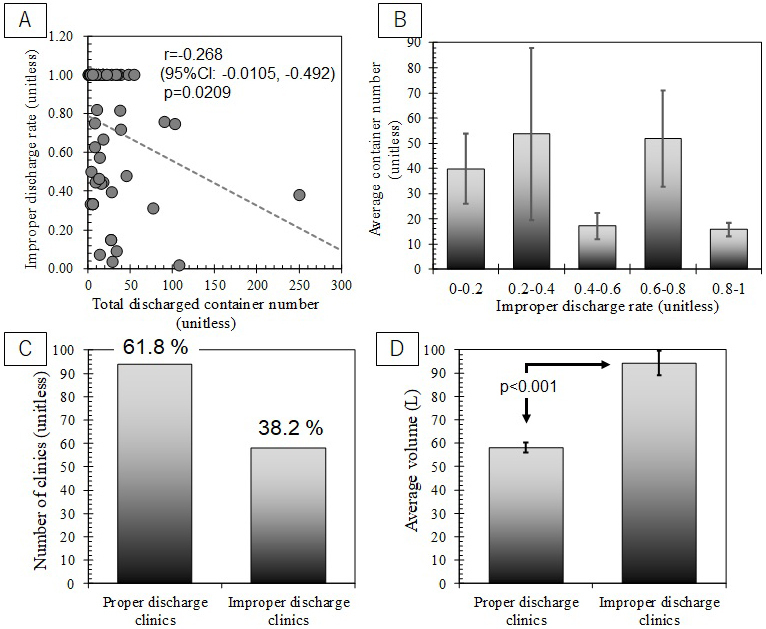
(a) Comparison between total discharged container numbers and improper discharge rate in each medical institution, (b) Average number of discharged containers from medical institutions with different improper discharge rates (c) The numbers of “proper discharge” and “improper discharge” medical institutions, and (d) Average waste volume discharged from “proper discharge” and “improper discharge” medical institutions (Note: p value of t-test for statistical significance of the correlation).

As described in the hypothesis sub-section, the first hypothesis of improper discharge mechanism is that longer interval of container discharge might induce less attentions toward proper discharge and cause human errors in disposal steps due to forgetting, incaution, or unwariness. This hypothesis expects that a moderate or strong negative correlation between improper discharge rate and container discharge frequency. Since container discharge frequency is proportional to total container discharge number, it was compared with improper discharge rate as shown in Figure 6a and 6b. Figure 6a clearly shows that there is weak correlation between improper discharge rates and container discharge numbers (r = −0.268). Even upper limit of 95% confidence interval is smaller than -0.500. No clear trend was also shown in Figure 6b. Therefore, this study found no inspectional evidence to support the first hypothesis.

### Second hypothesis validation: Does decreasing discharge cost induce overpacking and subsequent problems?

The second hypothesis is that decreasing discharge cost eventually causes overpacking of RMW. Larger capacity containers might induce overpacking more than small capacity containers. Subsequently, overpacking might cause improper sealing and container deformation. Therefore, the second hypothesis expects that improperly discharged RMW containers should have larger volume capacity than that of properly discharged containers. As shown in Figure 4a, 4b and 4c, the inspection results agree with the expectation. Significant differences were found in all cases. To validate significant association between overweight and container deformation, the inspection results are summarized in a cross table (see Table 4). Fisher’s exact test suggests significant associations between overweight and container deformation for 40 L containers (p = 0.00277) and 50 L containers (p < 0.001). 95% confidence interval of odds ratio is 11.6-199. This means that deformation probability is increased by 11.6-199 times when the containers are overweighted. The results of this study concluded that decreasing discharge cost induced overpacking more frequently in larger volume containers. Subsequently, it likely causes improper sealing, container deformation, and overweight.

**Table 4.** Cross Table of Container Numbers between Container Deformation and Overweight.

Container weight	Volume	No deformation				Container deformation			
		20 L (cuboid)	20 L (cube)	40 L	50 L	20 L (cuboid)	20 L (cube)	40 L	50 L
Regular weight	20 L (cuboid)	170	-	-	-	2	-	-	-
	20 L (cube)	-	1374	-	-	-	127	-	-
	40 L	-	-	315	-	-	-	16	-
	50 L	-	-	-	162	-	-	-	118
Over-weight	20 L (cuboid)	0	-	-	-	0	-	-	-
	20 L (cube)	-	0	-	-	-	0	-	-
	40 L	-	-	0	-	-	-	2	-
	50 L	-	-	-	2	-	-	-	70

### Third hypothesis validation: Does necessary compressive force for perfect sealing of a container lid induce improper sealing?

Figure 6c shows the number of small-scale medical institutions, which always properly discharged RMW containers, caused improper cases at least once or more. 61.8% of small-scale medical institutions perfectly discharged RMW containers. Meanwhile, 38.2% of medical institutions induced improper discharges. As shown in Figure 3c, 37.7% of RMW containers were categorized improper discharge on container number basis. There is good agreement between the percentage of “improper discharge” containers and that of medical institutions with “improper discharge.” It implies that improper discharges might not be sporadic events, which occurs in any medical institutions; whereas, it suggests that certain medical institutions likely repeat improper discharges. This result disagrees with the expectation from the third hypothesis. Figure 5 presents that the gender of clinic staff who handles RMW container discharges might be associated with improper sealing due to smaller compressive force. It might contribute into repeated partially improper discharges. Additionally, medical institutions with improper discharges generated larger volume of RMW than that of “proper discharge” medical institutions (see Figure 6d). It supports the second hypothesis. It shall be concerned socially that improper discharges might be repeated in certain clinics. Further researches to validate the recidivation are recommended strongly.

### Concluding summary and recommendations

As summary, the inspectional and statistical analysis support the second hypothesis but reject the first and third hypotheses. The second hypothesis proposes that saving RMW discharge cost might be the major factor of improper discharges. In decreasing discharge cost, overpacking occurs more frequently in larger volume containers and induces improper sealing, container deformation, and subsequently overweight. However, it should be noted that other factors like limited time of discharging waste might be also non-negligible for overpacking inducement. Less frequency of RMW container discharges and necessary compressive force for proper sealing are rejected to explain improper discharge mechanisms. Since improper discharges are considered to be repeated events in certain medical institutions, official monitoring to such institutions, in compliance to 80% fulfill recommendation to container volume capacity ^(1), (23)^, might be effective. The gender of clinic staff who handles RMW discharge is likely associated with improper sealing. Further survey to validate the proposal of “repeated improper discharge” is strongly recommended. In particular, it should be answered what causes the recidivation like less perception toward discharge appropriateness, busy working conditions of clinic staff, and/or others using well-designed questionnaire or interviews. They will be also conducive to clarify improper discharge mechanisms of RMW for efficient improvement of RMW management.

## Article Information

### Conflicts of Interest

None

### Author Contributions

Daisuke Sugimoto: Investigation and Writing - Original Draft.: Fumitake Takahashi: Conceptualization, Formal analysis, Writing - Review & Editing, and Supervision.

### Approval by Institutional Review Board (IRB)

This research does not require any ethical reviews and approvals because the research objects are the containers for RMW discharge, not medical waste. The authors had no accessibility to the medical wastes inside the containers and any patient information because re-opening of prohibited statutorily after they were sealed. All inspection data are owned by the authors for statistical analysis and publication.

## Supplement

Supplementary Materials
