# Supplementary material for: Motivation to Decrease Discharge Cost Results in Improper Discharge of Regulated Medical Wastes from Small Clinics: Inspectional and Statistical Evidence in the Tokyo Metropolitan Area
Source: JMA J. 2023 Apr 10;6(2):138–47. doi: 10.31662/jmaj.2022-0174 (PMC10169273; doi:10.31662/jmaj.2022-0174)
Supplement: Supplementary Materials [file 2433-3298-6-2-0138-s001.pdf]

Supplementary materials

Table S1 The number and location of inspected small-scale medical institutions, the number and volume of RMW containers

Category	Total number	Location and number	Inspected container number (-)	Total container volume (L)
Clinic	131	Tokyo (123), Kanagawa (5), Saitama (2), Chiba (1)	2089	53457
Veterinarians	12	Tokyo (9), Kanagawa (0), Saitama (1), Chiba (2)	244	9700
Dentists	3	Tokyo (3), Kanagawa (0), Saitama (0), Chiba (0)	7	140
Nursing homes	6	Tokyo (5), Kanagawa (1), Saitama (0), Chiba (0)	24	1020
Total	152		2364	64317

Table S2 Cross table of container numbers between container deformation and overweight

Container weight appropriateness	Volume	No deformation				Container deformation			
		20 L (cuboid)	20 L (cube)	40 L	50 L	20 L (cuboid)	20 L (cube)	40 L	50 L
Regular weight	20 L (cuboid)	170	-	-	-	2	-	-	-
	20 L (cube)	-	1374	-	-	-	127	-	-
	40 L	-	-	315	-	-	-	16	-
	50 L	-	-	-	162	-	-	-	118
Overweight	20 L (cuboid)	0	-	-	-	0	-	-	-
	20 L (cube)	-	0	-	-	-	0	-	-
	40 L	-	-	0	-	-	-	2	-
	50 L	-	-	-	2	-	-	-	70
